# Predatory nursing journals: A case study of author prevalence and characteristics

**DOI:** 10.1177/0969733020968215

**Published:** 2020-12-03

**Authors:** Sebastian Gabrielsson, Stefan Eriksson, Tove Godskesen

**Affiliations:** 5185 Luleå University of Technology, Sweden; 8097 Uppsala University, Sweden; 211737 Ersta Sköndal Bräcke University College, Sweden; Uppsala University, Sweden

**Keywords:** Academic publishing, predatory journals, publication ethics, research dissemination

## Abstract

**Background:**

Predatory publishing poses a fundamental threat to the development of nursing knowledge. Previous research has suggested that authors of papers published in predatory journals are mainly inexperienced researchers from low- and middle-income countries. Less attention has been paid to contributors from high-income countries.

**Aim:**

To describe the prevalence and characteristics of Swedish authors publishing in predatory nursing journals.

**Design:**

Quantitative descriptive case study.

**Participants and research context:**

Descriptive statistics were used to analyse the academic positions and academic affiliations of the authors of 39 papers published in predatory nursing journals during 2018 and 2019. Predatory nursing journals with Swedish contributors were identified by searching public listings of papers and applying a set of criteria. Journal site archives were used to identify additional papers with Swedish authors.

**Ethical considerations:**

This study was conducted in accordance with national regulations and ethical principles of research.

**Results:**

Almost two-thirds of Swedish authors publishing in predatory nursing journals hold senior academic positions. A small group of higher education institutions account for a majority of academic affiliations. Findings suggest that higher education institutions and experienced nursing researchers from Sweden make substantial contributions to predatory nursing journals, but that predatory publication habits might be concentrated in a limited number of academics and research milieus. A year-to-year comparison indicates that the prevalence of publishing in predatory journals might be diminishing.

**Discussion:**

Swedish nurse researchers help legitimize predatory journals, thus jeopardizing the trustworthiness of academic nursing knowledge. Substandard papers in predatory journals may pass as legitimate and be used to further academic careers. Experienced researchers are misleading junior colleagues, as joint publications might become embarrassments and liabilities.

**Conclusion:**

While the academic nursing community needs to address the problem of predatory publishing, there is some hope that educational efforts might have an effect on combating predatory publishing in nursing.

## Introduction

Due to technological advancements, the scholarly nursing community has seen the development of open access publishing challenging traditional ways of disseminating and accessing research findings. While profit-making might not be a phenomenon new to academic publishing, the rise of open access journals seem to have attracted gold-diggers with no interest in ensuring the trustworthiness of academic knowledge claims. Predatory journals and publishers are per definition ‘those that exploit the gold open-access model to profit from scholarly publishing in a dishonest way’ (p. 2).^1^ They can engage in unethical and unscholarly practices that typically involve ‘promises of rapid review and acceptance for publication, minimal to non-existent review processes, a fabricated editorial board, and mimicry of legitimate journal titles’ (p. 88).^2^ Although the nursing community have been warned about the perils of predatory publishing,^2^ researchers continue to contribute to predatory journals.

Research suggests that predatory publishing is mainly a concern for low- and middle-income countries. Shen and Björk^3^ reported a vast majority of corresponding authors from Asia (60.3%) and Africa (16.4%), with only 8.8% from Europe. Similarly, Xia et al.^4^ concluded that most authors who publish in predatory journals are young and inexperienced researchers from developing countries. Arguably, experienced researchers in high-income countries would be better prepared to refrain from contributing to predatory journals and expected to take responsibility and not legitimize these journals by lending them their names, titles and affiliations. Thus, All European Academies (ALLEA) have in their code of conduct for research integrity highlighted that to support ‘journals that undermine the quality control of research (‘predatory journals’)’ is an unacceptable practice.^5^ Nevertheless, scholars in Western high-income settings who have investigated the prevalence of predatory publishing in their own milieus have found that quite a number of colleagues have engaged with predatory journals.^6,7^

### Background

There are yet only a few studies on predatory publishing in nursing. Oermann et al.^8^ collected data on the characteristics of predatory nursing journals and surveyed authors, reviewers and editors about their experiences. They identified 140 predatory nursing journals from 75 publishers, and 4238 published papers, with a majority of the authors and editorial board members from India. Many of these short-lived journals typically lacked a focus on the particular research area of nursing, accepting papers on fringe subjects or whole other scholarly subjects. Survey answers described a variation of positive and negative experiences.^8^ Lewinski and Oermann^9^ examined the characteristics of e-mail solicitations that were sent from predatory nursing journals and identified flattering language, close due dates for submissions, general topics, and awkward phrases and incorrect word use as typical. Oermann et al.^10^ reviewed predatory nursing papers and found a lack of quality indicating poor peer review and editorial processes. Edie and Conklin^11^ tested the legitimacy of the peer review process and suggested that quality indicators related to language use, author control and transparency are good indicators for which journals to avoid. Owens and Nicoll^12^ compared the content of three different predatory nursing journals and found a substantial level of plagiarism and duplicate publications between them. Oermann et al.^13,14^ studied citation patterns and found that papers in predatory nursing journals are cited in non-predatory nursing journals.

### Aim

Predatory publishing poses a fundamental threat to the development of nursing knowledge. Previous research suggests that authors of papers in predatory journals are mainly inexperienced researchers from low- and middle-income countries. To assess this claim, the aim of this case study was to describe the prevalence and characteristics of Swedish authors publishing in predatory nursing journals, representative of a high-income country with a strong academic sector.

## Method

This study used a quantitative descriptive design. All data were retrieved from publicly accessible websites. Data for 2018 were collected in April and May 2019. Data for 2019 were collected in July 2020.

### Context

The Swedish context was chosen based on the authors’ familiarity with Swedish nursing research and researchers and because it is a suitable object for a case study. Sweden is a high-income European country with 10 million inhabitants in 2017.^15^ Nursing research is a well-established area of scholarship. There is nursing education on first-, second- and third-cycle levels at higher education institutions (HEIs). Universities have the entitlement to award first-, second- and third-cycle qualifications, and university colleges are entitled to award first- and second-cycle qualifications.^16^ In 2020, there are nursing education programmes at 25 HEIs, including both universities and university colleges. In June 2020, the Swedish Society of Nursing’s collection of doctoral dissertations contained 1832 titles, beginning in 1978, and the network for Swedish professors in nursing included around 100 members.^17^ One study found that an average of 0.5%–0.9% of the total publications from Swedish HEIs were published in questionable journals during the years 2012–2017, with a disproportionate large number of papers in nursing journals.^18^

### Data collection and sample

Despite sometimes claiming the opposite, predatory nursing journals are typically not indexed in bibliographic databases such as PubMed and CINAHL and may therefore not be discoverable through a traditional search.^8^ To avoid bias due to assumptions of what constitutes a predatory journal and who chooses to contribute to them, a systematic approach was applied to identify Swedish contributors to predatory nursing journals:

1. The first and third authors searched public listings of papers affiliated to Swedish HEIs to identify all papers classified as pertaining to nursing during 2018 and 2019. The main source was the DiVA portal which is a search tool for research papers written at 49 Swedish institutions (www.diva-portal.org), including most of the 25 HEIs involved in nursing education. Additional searches were made in local public listings of two large and one mid-sized HEI not included in the DiVA database. After removing duplicates and papers in non-English journals, this generated 652 unique records for 2018. The same procedure for 2019 generated an additional 717 unique records.
2. Journals in which these papers were published were identified, and the publishers of these journals were identified through information on journal websites. Publishers and journals were identified as predatory using distinct criteria (see below), generating a list of predatory nursing journals (n = 9) and associated papers (n = 15) with Swedish authors published in these journals in 2018. A manual search of these journals publication archives identified additional papers with Swedish authors (n = 15). The same procedure for 2019 yielded yet additional papers (n = 9).
3. We also searched Cabells blacklist of predatory journals (www2.cabells.com) to identify additional predatory nursing journals. A manual search of the publication archives of these journals identified additional journals (n = 4) and papers (n = 10) with Swedish authors. However, these were identified as hijacked papers (see below) and excluded from our study.
4. All identified papers published in predatory nursing journals during 2018–2019 with at least one Swedish affiliation were retrieved in full text.

This systematic search process generated a sample of 39 papers with at least one author affiliated with a Swedish HEI that were published in nine predatory nursing journals published by seven predatory publishers.

#### Identification of journals as predatory

To identify from established criteria those journals that could count as predatory, we consulted renowned blacklists and whitelists. The Cabells blacklist (now changed to ‘predatory reports’) is a commercial service using a team of specialists to ‘analyze over 60 behavioral indicators [called “violations”] to keep the community aware of the growing threats and to keep academia protected from exploitative operations’.^19^ Cancelled in 2017, the previously available Beall’s list was a blacklist of predatory journals and publishers to be avoided.^20^ However, the list was criticized for a lack of transparency and thus reliability, and since its removal it has been argued that it is going out of date.^21^ For this study, we primarily used Cabells’s more developed list in combination with two well-known journal ‘whitelists’: the Committee on Publications Ethics (COPE) member directory (https://publicationethics.org), and the Directory of Open Access Journals (DOAJ) (https://doaj.org/). Journals were classified as predatory if

- the publisher was listed as predatory by Cabells or, in two cases, by Beall, and
- the journal was not listed in the COPE member directory, and
- the journal, if fully open access, was not listed in the DOAJ.

Regarding the two journals listed in Beall’s list but not Cabells, they have both been heavily criticized for being predatory and were in this respect well known to the authors of this study. The journals listed on Cabells and included in this study had each a reported number of violations ranging from 4 to 11. Criticism reported on Internet regarding the two journals listed by Beall shows that there *at least* are reports of four behavioural indications or violations for each of those journals; therefore, they were included in this study. It should be noted that one publisher included from Cabells was under review there, and that the results have not yet been published. As for the two Beall’s inclusions, this one journal clearly has more than four violations, based on published criticism.

#### Exclusion of hijacked papers

The authors excluded 10 abstracts from this study, found in four predatory journals. A closer look at them revealed that all these 2019 abstracts came from papers previously published in legitimate journals and that just one particular publisher was to blame. It seems that these papers were lifted verbatim from public archives, not submitted by the authors themselves, and therefore not suitable for inclusion here.

### Analysis

Descriptive statistics were used to analyse author characteristics. Statistical analysis was done using Excel. For the purpose of this study, we differentiated between *authors* and *authorships*. An *author* is considered a unique individual who is listed as an author of one or more papers. An *authorship* is the listing of a unique individual as an author of a paper. Thus, one author can hold many authorships. The two variables in focus were academic positions and academic affiliations. In addition to author information in the papers and on journal websites, information about academic positions was retrieved from HEI websites. Academic positions were grouped according to the main employment categories used in Swedish HEIs. Academic affiliations were grouped according to HEI and further classified as small, mid-size or large (Table 1). For the purpose of this analysis, a classification was made depending on the total number of doctoral students enrolled at the HEI,^22^ not the number of nursing doctoral students. This basis for classification was chosen because it was deemed manageable, yet likely to be relevant for the research milieus in HEIs.

**Table 1.** Classification of higher education institutions (HEIs) according to number of doctoral students.

Number of students	Size
<500	Small
500–1500	Mid-size
>1500	Large

### Ethical considerations

As this study made use of only non-sensitive data, no formal ethical approval is required according to the Swedish Act on Ethical Review.^23^ The study does recognize the importance of adhering to the principle of confidentiality when processing personal data. Therefore, all data have been kept secure and all the names of journals, authors and affiliations have been omitted from the presentation of the findings, as the issue here is what characterizes Swedish authors of papers in predatory journals, not who they are or where they work or which predatory journal managed to ensnare them.

## Results

Authors were classified according to academic positions (Table 2). The total number of individual authors was 84, while the total number of authorships was 108. Close to two-thirds (61.9%) of the authors claiming Swedish affiliations held senior academic positions, as professors or senior lecturers. A smaller group (10.2%) held junior positions, as either PhD students or lecturers. A quarter (26.1%) of authors could not be identified as holding academic positions through HEI websites.

**Table 2.** Academic positions of authors and authorships with Swedish affiliations publishing in predatory nursing journals in 2018–2019.

	Authors		Authorships	
	n	%	n	%
Professor	14	16.7	19	17.6
Senior lecturer	38	45.2	54	50.0
Lecturer	3	3.6	4	3.7
PhD student	7	8.3	7	6.5
Not known	22	26.1	24	22.2
Total	84	99.9	108	100.0

Thirteen authors had authored more than one paper and, with the exception of one lecturer and two authors whose academic position could not be identified, these held senior positions as professors (n = 3) or senior lecturers (n = 7). One of these authors had had seven papers published. Eight authors had published both in 2018 and 2019, of these seven held senior academic positions. Almost half of authorships (47.2%) involved a senior researcher as first (n = 20), last (n = 22), corresponding (n = 25) or single (n = 5) author.

Fourteen authors were professors. Ten of these were professors in nursing or caring science. Five were professors’ emeriti. Seven were affiliated with small HEIs, five with large HEIs and two with mid-sized HEIs.

**Figure 1.**
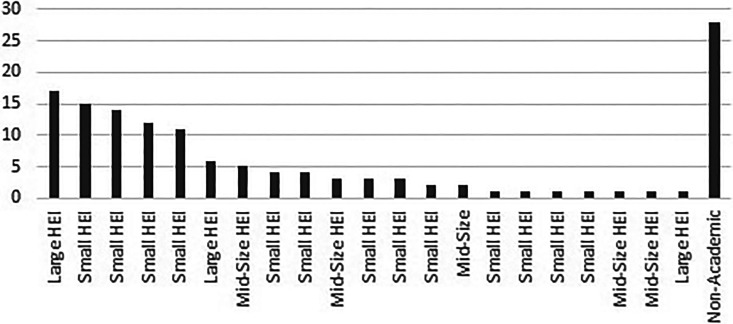
Number of claims of Swedish affiliations (n = 136) in papers published in predatory nursing journals 2018–2019 per affiliation.

Figure 1 illustrates the distribution of authors claims of affiliations (n = 136) among Swedish institutions. A small group of HEIs (n = 5) accounted for a majority (63.4%) of academic affiliations (n = 108) claimed in the papers.

**Figure 2.**
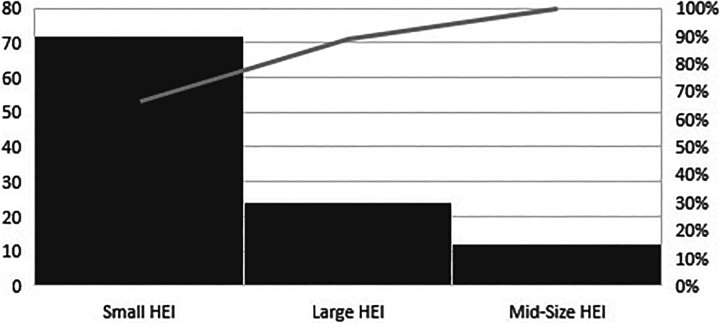
Distribution of Swedish academic author affiliations claimed in predatory nursing publications 2018–2019 (n = 108) according to size of higher education institution.

Figure 2 illustrates the distribution of academic affiliations according to the size of the HEI. Small HEIs account for two-thirds (66.7%) of academic affiliations.

A total of 39 papers were published during 2018 and 2019. In 2019, the total number of publications had decreased to 9 papers from 30 the year before.

## Discussion

While previous research suggested that contributors to predatory journals are mainly inexperienced researchers from low- and middle-income countries,^3,4^ our study offers a complementary perspective as it identified 39 Swedish-authored papers published in nine predatory nursing journals. Compared to the number of nursing papers retrieved in the database search (2018 n = 652; 2019 n = 717), the existence of 39 papers in predatory journals suggests that predatory publishing might not be a fringe phenomenon in the Swedish nursing context (comprising 2.8%). This confirms previous research.^18^ So, then what can be learned from the case of Sweden? A number of ethical issues affecting nursing are noteworthy, since we have found quite a significant amount of papers published in predatory journals by senior academics.

For a start, this is highly problematic as those senior scholars, by submitting papers to predatory nursing journals, help *legitimize* predatory publishing, thus jeopardizing the trustworthiness of academic nursing knowledge. Nursing scholars will look at the authors previously having published with a journal; finding senior colleagues from Sweden among them can be seen as an indicator of journal quality. This might blur the line between legitimate and predatory journals over time, making it increasingly hard to uphold the distinction.^24^

Then there is the *injustice* affecting the nursing community; in that some scholars are using a fast track publishing route to get ahead, sometimes at the expense of colleagues.^25^ Nursing scholars undertaking peer review or serving on examination or employment boards need to be aware that publications cited in research papers or claimed in applications for research funding and academic positions might be published in predatory journals and that they should be judged accordingly.

A further ethical issue is that the seniors risk influence the publication practices of junior researchers. Of the 108 authorships related to papers appearing in predatory nursing journals, almost two-thirds of the authors claiming academic affiliations held senior academic positions, including 10 nursing professors. This is not surprising considering that senior researchers in general might be expected to be more prolific than their juniors. On the other hand, senior researchers might also be expected to be better able to identify dubious journals and take a greater responsibility in fostering sound publishing practices. Instead, juniors following suit might believe that journals offering just cursory review and a quick route to publication are laudable for their ‘effectiveness’ and that it is okay to publish with them. The seniors should rather act as role models and safeguards by being vigilant and setting standards as to what constitute acceptable channels for dissemination of research findings.

It is also interesting that our results put a light on a new form of predatory practices, that is, the hijacking of articles. The existence of hijacked journals has received some attention^26–28^ but this phenomenon is not as well known. Still, it seems noteworthy that there are 10 instances of this in 2019, while there were none in 2018; publishers and authors will now likely have to guard themselves against such misappropriation. As to why this practice occurs, hijacking papers in this way may serve the purpose of making it look like the journals publish papers by renowned scholars, in order to attract academic authors. We have chosen to report the responsible publisher to the two main copyright holders of those hijacked papers.

Last, our results invoke a question of how to handle findings such as ours. We have chosen to react against the hijacking of articles, as noted, but keep the identities of the Swedish authors and HEIs confidential. Is this what should be done? If a scholar browsing article indexes finds colleagues who publish extensively or repeatedly in predatory journals, should they then not blow the whistle? The All European Academies (ALLEA) guideline condemns support of predatory journals as an *unacceptable practice*, which seems to indicate that we should not accept such deviations from good practice. On the other hand, reporting it might result in honest mistakes being taken as intentional misconduct, it might be destructive to research relations, and it can result in a questionable culture of squealing. No surprise then that present ways of handling cases often strike us as awkward. This study points to the need for further ethical deliberation over how to proceed when finding predatory publishing in our own academic nursing settings; the stakes are significant.

One aspect of this dilemma is that authors who perhaps have intentionally chosen to publish with predatory journals might also deviate from good research practices in other ways as well.

We do not know why the authors in this study chose to publish in predatory nursing journals. When Oermann et al.^8^ surveyed authors, some insisted that they were familiar with the journals and that they were reputable, while others said they had simply responded to e-mail invitations or followed the recommendations of colleagues. Possible explanations could include a lack of awareness about predatory publishing, opposition to labelling open access or non-conformative journals and publishers ‘predatory’, an indifference to the negative consequences of predatory publishing, or just that these journals provide the services that authors ask for.^7,29,30^ In any case, it is reasonable to be suspicious about an author’s research output when said author publishes in a predatory journal; to choose such an outlet just *might* serve the purpose of concealing plagiarism, data manipulation or other breaches of good practice. One could argue that it is important therefore to expose those engaging in predatory practices, even if this would not in itself be considered a matter of serious misconduct; as such a practice might conceal more deviations that are serious.

There is a potentially *positive* finding in this study. While outside the scope of our study, when researching authors’ HEI websites, it appeared to be the case that at least a few senior researchers do publish in predatory journals on a more regular basis, as we saw examples of papers published both before and after 2018–2019 and in predatory journals not classified as nursing journals. While publishing in predatory journals might initially benefit authors, to do so might come to damage a researcher’s reputation. In the words of the InterAcademy Partnership (IAP), it is increasingly realized that predatory practices are ‘threatening to cause widespread, long-term damage to knowledge generation, academic integrity and the research enterprise as a whole’.^31^ Since this study comprises a 2-year period, it is nevertheless possible to see that the number of Swedish publications in the investigated journals rapidly diminished from 30 in 2018 to 9 in 2019. These findings align with a previous general decline in questionable publishing in Sweden, as reported by Nelhans and Bodin.^18^ During this time and in the previous few years, there have been a number of papers addressing predatory publishing in the academic press, seminars discussing it at medical faculties, the subject has been included in nurse education, and quite a few cases at HIEs have highlighted the importance of being resilient to the lure of predators. It is possible that these measures have had an effect reflected in our result. This underlines that we should make an effort to ensure that all nurses about to submit manuscripts for academic publishing are aware of the characteristics of predatory journals and able to take reasonable precautions; to check journals against reputable whitelists (e.g. COPE and DOAJ), blacklists (e.g. Cabells), or use assessment tools as the Journal Evaluation Tool^32^ and the Think.Check.Submit. checklist.^33^

## Limitations and need for further research

The comparison between the output of nursing research and the number of predatory papers in Sweden must be made with caution as only predatory nursing journals were subject to the additional manual search, adding to the number of predatory nursing journals identified. If additional papers in non-nursing and non-predatory journals had also been sought manually, it is possible that the proportion of papers published in predatory nursing journals could have decreased. However, it is also possible that papers in predatory journals have been intentionally omitted from HEIs’ public listings, due to quality control measures or authors realizing the dubious nature of journals not wanting to display such papers.

Caution should also be exercised regarding the extent as to which predatory publishing is practised intentionally. The results show that 13 authors – all but three senior academics – had published more than one paper in such journals. As this was a descriptive study limited to 2018–2019, it is not possible to conclude that individual researchers have adopted predatory publishing as a conscious strategy. We also hypothesize that efforts to inform and educate might have diminished the number of predatory publication from Swedish nursing authors, but this is conjecture. Further research might apply a longitudinal design to observe publication practices over time.

In this study, a small group of academic institutions accounted for a majority of the academic affiliations claimed in the analysed papers. Small HEIs accounted for two-thirds of the academic affiliations. These results indicate that, at least in Sweden, predatory nursing publishing might be mainly concentrated in a few smaller research milieus, suggesting differences in peer review and support, academic culture, leadership and/or competence. It is also worth noting the substantial number of authors lacking academic affiliations and/or academic positions. Possibly, these represent clinically employed nurses and other healthcare professionals involved in research and development. Further research is needed to understand how research milieus might hinder or enable predatory publishing.

## Conclusion

This article demonstrates that predatory publishing is not limited to low- and middle-income countries or junior researchers. Experienced researchers in high-income countries also engage in predatory publishing. Although not isolated to them, small research environments might be especially vulnerable for the development of poor dissemination practices, making them easier prey for predatory publishers. The results of this study should worry nursing scholars. Senior nurse academics need to honour the trust bestowed upon them by the scholarly nursing community and take the lead in ensuring the rigour and trustworthiness of nursing knowledge. While the problem of predatory publishing needs to be continuously addressed by the academic nursing community, there is some hope from this study that educational efforts might have an effect in combating predatory publishing in nursing.
